# COVID-19: the implications and consequences of prolonged lockdown and COVID-19 vaccine cost in a low-middle income country

**DOI:** 10.11604/pamj.2021.39.48.27674

**Published:** 2021-05-19

**Authors:** Akaninyene Eseme Ubom, Omotade Adebimpe Ijarotimi, Solomon Nyeche, John Igemo Ikimalo

**Affiliations:** 1 Department of Obstetrics, Gynaecology and Perinatology, Obafemi Awolowo University Teaching Hospitals Complex, Ile-Ife, Osun State, Nigeria,; 2 Department of Obstetrics, Gynaecology and Perinatology, Faculty of Clinical Sciences, College of Health Sciences, Obafemi Awolowo University, Ile-Ife, Osun State, Nigeria,; 3 Department of Obstetrics and Gynaecology, University of Port Harcourt/University of Port Harcourt Teaching Hospital, Port Harcourt, Rivers State, Nigeria

**Keywords:** COVID-19, vaccine, cost, lockdown, neglected tropical diseases, lassa fever

## Abstract

Lockdowns and just recently, the COVID-19 vaccines, are amongst the disease containment measures instituted globally to check the spread of COVID-19. Prolonged lockdowns are however, not sustainable in low resource economies like Nigeria, where up to 70% of her population live on less than a dollar a day, with the majority, either unemployed, or working in the private/informal sector and depending on daily earnings for survival. If the lockdown remains sustained, it would not be long before the largely poor citizens starve to death. Also, spending over US $3.9 billion on COVID-19 vaccines for more than 200 million Nigerians, as intended by the Nigerian government, is not plausible, given that neglected tropical diseases (NTDs) like Lassa fever, and other more common causes of morbidity and mortality, continue to kill more Nigerians than COVID-19. Public enlightenment of the populace on the need to strictly adhere to non-pharmacologic preventive measures, including social distancing, use of face masks, good personal hygiene, covering of the mouth and nose when coughing and sneezing, frequent hand washing and sanitizing with alcohol-based hand-sanitizers and disinfection of surfaces, is what is sustainable, feasible and compatible with the economic reality in our setting. As Sir Robert Hutchison, the highly revered doyen of medicine, wrote in his petition over 85 years ago, “And from making the cure of the disease more grievous than the endurance of the same, Good Lord, deliver us”, we must be careful not to make the cure of COVID-19 worse than COVID-19 itself.

## Commentary

The novel coronavirus disease-2019 (COVID-19) broke out in Wuhan, Hubei Province, China, in late December 2019. Since its emergence, the disease has spread to 221 countries and territories worldwide, with over 75 million infections and more than 1,500,000 related deaths, making it the most devastating pandemic in the last 100 years, after the Spanish flu [1]. The traditional public health measures presently instituted worldwide to contain the disease include social distancing, frequent handwashing and sanitizing with alcohol-based hand sanitizers, isolation, quarantine, lockdowns and just recently, the COVID-19 vaccines [1].

In Nigeria, the government declared a total lockdown on 30^th^ March 2020 [2]. Though this was relaxed on 27^th^ April 2020, to allow for minimal essential activities, disease containment measures like curfew, restriction of work hours and social gatherings continued to be in force and on 21^st^ December 2020, the Nigerian government in response to the second wave of the pandemic, issued new restrictions to check the spread of the virus [2,3]. These new restrictions include the immediate closure of all restaurants, night clubs, bars, pubs, event centres and other recreational venues across the country for five weeks [3]. Schools have been shut until January 18^th^, 2021, just as all federal civil servants on grade levels 12 and below, are to work from home for five weeks [3]. With the fresh restrictions, the country continues to be under lockdown for nine months now and still counting.

The untold economic hardship suffered by citizens and families due to the prolonged lockdown has far reaching consequences and implications, especially so in a country like ours, where up to one-half of her population live below poverty line, with the majority either unemployed or working in the private/non-formal sector [4]. For these ones, the lockdown denies them the daily earnings that they survive on, as shops and businesses that do not provide essential services remained closed during the lockdown. Several jobs in the private/informal sector have been lost to downsizing and workers´ salaries slashed, as firms are no longer able to maintain staff overhead costs [4]. Food prices have skyrocketed and transportation costs, increased, owing to the fact that public vehicles are now mandated to carry passengers at 50 percent of their full capacity per time, in compliance with social distancing measures. A fallout of these has been an increase in criminal activities, especially in large cities like Lagos, a hotbed of the disease in Nigeria [4].

On a national scale, with the fall in global oil prices, precipitated by the pandemic and the consequent drop in Nigeria´s net oil revenue, the Nigerian economy faces collapse [5]. Borrowers´ capability to service their loans have been drastically affected, resulting in a rise in non-performing loans, and inability of banks to grant additional loans to small and medium enterprises (SMEs), that account for over 90% and 80% of businesses and employment respectively, in Nigeria [5]. Recruitment into the non-essential public sector in the country has remained frozen for lack of funds. The Nigerian stock market is crashing daily, even as some state governments are either no longer able to pay workers´ salaries, or are struggling to pay a fraction of the salaries, due to a drop in federal allocation to these states, following a downturn in crude oil sales [5]. At some point, three months into the pandemic, even the federal government was unable to pay resident doctors in the country the negotiated COVID-19 hazard allowances and this led to a nation-wide industrial action by the resident doctors, which crippled the health care sector for a week. The government has been able to pay these allowances for only three months.

Furthermore, owing to the economic challenges posed by the COVID-19 pandemic, Nigeria´s 2021 budget incorporates a deficit of about ₦5.20 trillion, which is approximately 3.64% of gross domestic product (GDP), a figure that is above the 3% benchmark set by the Fiscal Responsibility Act. At this rate, the Nigerian federal government may no longer be able to sustain its current stimulus packages to small businesses and state governments in the country, to cushion the economic effects of the lockdown. Away from the economy, education has also been negatively affected by the pandemic. All tertiary institutions in the country have been shut since 19^th^ March 2020, even before the lockdown. The psychological impact of a disrupted academic calendar and the stall in career progression/graduation on undergraduates can be untoward. With the closure of tertiary institutions, medical schools in public universities in the country, have neither admitted fresh medical students nor graduated medical doctors since the lockdown began. This has the potential of further worsening Nigeria´s current very low physician-to-patient ratio of four doctors per 10,000 patients. Only a few private universities have continued to lecture their students using online technologies [6]. However, with the high cost of internet in the country and poor mobile networks [2], sustaining online education is challenging.

Healthcare delivery and patient care is not left out of the adverse effects of the lockdown. The closure of outpatient clinics and postponement of elective surgical operations, in line with lockdown measures, saw a significant rise in morbidity and mortality from various disease conditions. The lockdown also significantly impacted postgraduate medical training in the country, especially in the surgical specialties, that largely depend on patient flow for hands-on experience [2]. The two postgraduate medical/surgical colleges in the country (the National Postgraduate Medical College of Nigeria and the West African College of Surgeons/Physicians) had to postpone the March/April/May 2020 Part 1/Membership and Part 2/Fellowship examinations for six months, due to the pandemic.

The Nigerian health minister recently announced plans to procure 200 million doses of the COVID-19 vaccines for Nigerians by January 2021 [7]. This would cost the government up to US $3.9 billion [8], more than a tenth of Nigeria´s total budget of about US $34.2 billion for 2021. Even this amount is enough to vaccinate only about 50% of Nigeria´s total population of over 200 million, as the currently available vaccines require two doses for full protection. According to experts, at least 60% of the population must be vaccinated to achieve herd immunity. If this is the case, then the government would need to spend even more on the vaccines before the desired herd immunity is achieved.

Expending so much on COVID-19 vaccines when neglected tropical diseases (NTDs) like Lassa fever, which is endemic in Nigeria, continue to kill more Nigerians than COVID-19, does not seem plausible. Since Nigeria confirmed her first case of COVID-19 on 27^th^ February 2020, she has to date, recorded about 75,000 cases and 1,200 related deaths [7] (CFR of 1.6%). On the other hand, in 2020, 1,175 confirmed cases of Lassa fever were reported in 27 states and 130 local government areas in Nigeria (an upsurge from 817 in 2019), with 242 deaths, giving a CFR of 20.6% [9], compared to 1.6% for COVID-19, yet Lassa fever is not given as much attention and response as COVID-19. This further underscores the fact that the current focus on COVID-19 has significantly distracted attention from more common and known causes of morbidity and mortality in Nigeria like malaria, pneumonia, diarrhea, road traffic accidents, heart diseases, obstructed labour and other pregnancy complications, amongst others, and this has led to an increase in deaths from other causes unrelated to COVID-19.

The amount of funds to be spent on procuring COVID-19 vaccines would be better utilized if used to fund the Nigeria Centre for Disease Control (NCDC), the country´s national public health institute, to fight NTDs like Lassa fever, and other endemic diseases, that continue to kill more Nigerians than COVID-19. Such monies should be used to fund research and development of vaccines for these deadlier endemic diseases than COVID-19. The foregoing said, it is increasingly becoming apparent that COVID-19 is here to stay, and we will have to live with it for a while. Continuous lockdown, while the country daily plunges further into economic recession, no longer seems reasonable, and neither is it sustainable. We definitely cannot keep our economy, schools, national and daily lives locked down indefinitely. If we do, it would not be long before the cure of the disease becomes worse, and kill more than the disease itself. The mostly poor masses would starve to death if the country stays locked down for too long further.

*"And from making the cure of the disease more grievous than the endurance of the same, Good Lord, deliver us"* [10], this petition by the highly revered doyen of medicine, Sir Robert Hutchison, written more than 85 years ago, as then, is as relevant now. A more sustainable, feasible and viable disease containment measure in our setting is massive public enlightenment of the populace on the need to strictly adhere to non-pharmacologic preventive measures, including social distancing, use of face masks, good personal hygiene, covering of the mouth and nose when coughing and sneezing, frequent hand washing and sanitizing with alcohol-based hand-sanitizers, and disinfection of surfaces.

## Conclusion

As the COVID-19 pandemic persists, continuously keeping a low-middle income country like Nigeria under lockdown is not sustainable, given the dire economic consequences of such prolonged lockdown. It is also not plausible to expend so much funds on COVID-19 vaccines, when NTDs like Lassa fever and other more common causes of morbidity and mortality, continue to kill more Nigerians than COVID-19.
